# Effect of the Drying Process on the Intensification of Phenolic Compounds Recovery from Grape Pomace Using Accelerated Solvent Extraction

**DOI:** 10.3390/ijms151018640

**Published:** 2014-10-15

**Authors:** Hiba N. Rajha, Walter Ziegler, Nicolas Louka, Zeina Hobaika, Eugene Vorobiev, Herbert G. Boechzelt, Richard G. Maroun

**Affiliations:** 1 Centre d’Analyses et de Recherche, UR TVA, Faculté des Sciences, Université Saint-Joseph, B.P. 11-514 Riad El Solh, Beirut 1107 2050, Lebanon; E-Mails: hiba.rajha@usj.edu.lb (H.N.R.); nicolas.louka@usj.edu.lb (N.L.); zeina.hobaika@usj.edu.lb (Z.H.); 2 UTC/ESCOM, EA 4297 TIMR, Département de Génie des Procédés Industriels, Laboratoire Transformations Intégrées de la Matière Renouvelable, Université de Technologie de Compiègne, Centre de Recherche de Royallieu, Compiègne Cedex, BP 20529 – 60205, France; E-Mail: eugene.vorobiev@utc.fr; 3 Institute of Chemistry, Karl-Franzens University of Graz, A-8010 Graz, Austria; E-Mails: walter.ziegler@uni-graz.at (W.Z.); herbert.boechzelt@joanneum.at (H.G.B.); 4 JOANNEUM RESEARCH Forschungsgesellschaft mbH, Institute RESOURCES, A-8010 Graz, Austria

**Keywords:** byproduct valorization, grape pomace, phenolic compounds, accelerated solvent extraction, scavenging activity

## Abstract

In light of their environmental and economic interests, food byproducts have been increasingly exploited and valorized for their richness in dietary fibers and antioxidants. Phenolic compounds are antioxidant bioactive molecules highly present in grape byproducts. Herein, the accelerated solvent extraction (ASE) of phenolic compounds from wet and dried grape pomace, at 45 °C, was conducted and the highest phenolic compounds yield (PCY) for wet (16.2 g GAE/100 g DM) and dry (7.28 g GAE/100 g DM) grape pomace extracts were obtained with 70% ethanol/water solvent at 140 °C. The PCY obtained from wet pomace was up to two times better compared to the dry byproduct and up to 15 times better compared to the same food matrices treated with conventional methods. With regard to Resveratrol, the corresponding dry pomace extract had a better free radical scavenging activity (49.12%) than the wet extract (39.8%). The drying pretreatment process seems to ameliorate the antiradical activity, especially when the extraction by ASE is performed at temperatures above 100 °C. HPLC-DAD analysis showed that the diversity of the flavonoid and the non-flavonoid compounds found in the extracts was seriously affected by the extraction temperature and the pretreatment of the raw material. This diversity seems to play a key role in the scavenging activity demonstrated by the extracts. Our results emphasize on ASE usage as a promising method for the preparation of highly concentrated and bioactive phenolic extracts that could be used in several industrial applications.

## 1. Introduction

The exploitation of food byproducts has been continuously increasing due to environmental and economic interests [1]. The value of food wastes is associated with their content in dietary fibers and bioactive molecules amongst which, antioxidant phenolic compounds [2], which can be capitalized in the food, pharmaceutical and cosmetic industries [3]. Several grape (*Vitis vinifera*) byproducts such as pomace are rich in antioxidant substances namely phenolic compounds [4]. These have several pharmaceutical and nutritional applications. Consisting of skins, seeds and stems, and representing approximately 20% (*w*/*w*; Fresh Weight) of the processed grapes weight, grape byproducts are abundantly obtained, especially considering that grapes are amongst the most cultivated crops in the world [5]. At an environmental level, problems related to grape pomace disposal could emerge when burying these byproducts, since this could affect the soil and the groundwater quality, and the flora and fauna. When used as fertilizers, they even might prevent germination properties. Consequently, the valorization of those byproducts reduces waste and permits the purification of added-value products. It follows that apart from their individual and public health benefits, phenolic compounds are highly valuable on an industrial level; therefore the main focus is on the environment friendly extraction methods of those molecules, using green technology by replacing the conventional methods with innovative technologies [6,7]. Accelerated Solvent Extraction (ASE), firstly described by Richter *et al.* (1996) [8], is one of the unconventional energy saving methods. It is an automated rapid extraction technique that utilizes common solvents at elevated temperature and pressure, and thereby increases the efficiency of extraction of organic compounds from solid and semisolid matrices. It allows extractions for sample sizes 1–100 g in minutes, reduces solvent uses dramatically, and can be applied to a wide range of matrices, including natural products. This technique is based on the heating process of the solvent (water or organic solvent) accompanied by a high pressure, which prevents the boiling phenomenon [9]. ASE allows the extraction of diverse compounds arising from different plants since the polarity and the temperature of the solvent can be changed to fit the adequate matrix [10]. Yet, the extraction at relatively high temperatures is likely to enhance the formation of furanic compounds (furfural, hydroxymethylfurfural), which are toxic products resulting from sugar degradation. The U.S Food and Drug Administration reported furans to be harmful substances in thermally heated food. However, Delgado-Torre *et al.* (2012) [11] studied the formation of hydroxymethylfurfural (HMF) by Superheated Liquid Extraction (SHLE) of polyphenols from vine shoots at temperatures varying from 160 to 240 °C. They found that the SHLE of polyphenols significantly increased the formation of HMF when processed at temperatures above 180 °C. Using ASE, Maillard and caramelization reactions might occur leading to the formation of new products. However, this was not studied in our work, since it was reported by Plaza *et al.* (2010) [12] that the complexity of the samples, which contain diverse compounds (other than amino acids and sugars) potentially affect the incidence of Maillard and caramelization reactions. As many factors are likely to significantly influence the efficacy of an extraction, the optimization of the process seems necessary. In order to achieve our goal we used in this study Response Surface Methodology (RSM), which is an assemblage of statistical and mathematical methods fruitfully used for the development, improvement, and design of processes [13]. One of the oldest essential features of food conservation and processing is drying. It refers to the elimination of moisture from a substance, mainly to diminish microbiological spoilage and increase shelf life [14,15,16].

The effect of the drying temperatures (60, 100, and 140 °C) on polyphenol content and antioxidant activity was studied for grape pomace peels. Drying at 60 °C did not affect the extraction of polyphenols nor their stability compared to freeze-dried samples [17]. Air-drying temperatures above 60 °C were reported to significantly decrease the radical scavenging activity and polyphenols levels in mulberry (*Morus alba* L.) leaves compared to freeze-dried samples [18].

In our study, the drying process was conducted on grape pomace (seeds and peels) at 45 °C. It was used as a pretreatment for the accelerated solvent extraction process, which was conducted at 40, 60, 80, 100, 120 and 140 °C. The effect of the drying pretreatment on the polyphenol extraction process and radical scavenging activity was studied. It was compared to untreated wet pomace extracts for further comprehension of the drying process. We were encouraged to use high temperatures (up to 140 °C) in this study, since heat treatment (up to 150 °C) was shown to increase the antioxidant activity of citrus peel extracts through the formation of low molecular weight phenolic compounds [19]. The objectives of this work were double; first we determined the experimental conditions (temperature and solvent mixture ratio) for total phenolic compounds extraction from grape byproducts using ASE in presence of green solvent mixtures and second we studied the effect of the drying process of the raw material on the quantity and quality of the resulting extracted compounds. Besides valorization of grape byproducts, the major aim of our work was to optimize the experimental parameters capable of reducing the energy cost of phenolic compounds extraction from grape pomace in order to produce natural antioxidant molecules with a high added value for several industrial applications. This study was designed to meet the Green Extraction concept. It fulfills the majority of the principles mentioned by Chemat *et al.* (2012) [20]. Grape pomaces are renewable plant resources. Their use as a source of natural products does not lead to the over-exploitation of grapes, since they are winemaking by-products. The valorization of grape pomace is likely to (1) heighten their economic interest; (2) reintegrate waste into the food cycle and (3) avoid serious environmental problems regarding their disposal. Moreover, ethanol was used as a solvent for the extraction process. It is a biodegradable bio-solvent generally recognized as safe (GRAS) by the U.S. Food and Drug Administration. The optimization by RSM and the use of an innovative extraction process “Accelerated Solvent Extraction” are likely to intensify the biomolecule production minimizing solvent consumption, energy, time and cost of the process.

## 2. Results and Discussion

### 2.1. Total Phenolic Content Optimization by Response Surface Methodology

RSM was revealed to be an influential tool in the optimization of experimental conditions to maximize various responses [21,22,23,24]. Herein, the effect of the temperature and the solvent mixture on the extraction of total phenolic compounds from wet grape pomace was studied in order to select the optimal conditions for the process. Once these conditions established they were applied for the rest of the study. Temperature and solvent mixture were the main parameters reported to affect the extraction of phenolic compounds from byproducts using accelerated solvent extraction [25]. Table 1 shows the 3-level central face composite design with three blocks and twelve runs (factorial design runs 1 to 4; axial points, runs 5 to 8 and central repetition points, runs 9 to 12). 

**Table 1:** Arrangement for independent variables and their responses for ASE process.

Run	Blocks	Variables Levels Uncoded (Coded)			
		**Temperature (°C)**	**Solvent Mixture (%etdanol/water)**	**Phenolic Compounds Yield (g GAE/100 g DM)**	
				**Experimental**	**Predicted**
1	1	60 (−1)	30 (−1)	6.02	6.23
2	1	60 (−1)	70 (+1)	9.94	9.94
3	1	140 (+1)	30 (−1)	11.62	11.62
4	1	140 (+1)	70 (+1)	16.31	16.1
5	2	60 (−1)	50 (0)	7.56	7.35
6	2	140 (+1)	50 (0)	13.72	13.93
7	2	100 (0)	30 (−1)	9.31	9.1
8	2	100 (0)	70 (+1)	12.18	12.39
9	3	100 (0)	50 (0)	10.50	10.49
10	3	100 (0)	50 (0)	10.68	10.49
11	3	100 (0)	50 (0)	10.64	10.49
12	3	100 (0)	50 (0)	10.18	10.469

In parallel, we did utilize a maximum of 70% ethanol content in order to propose a cost-effective and economical process. The values of the response variable Phenolic compounds yield (PCY), given as g Gallic Acid Equivalent (GAE)/100 g dry matter (DM), are shown for the experimental combinations of the variables. The closeness of the predicted values to the observed results shows good adequacy of the adopted model to the experiments. The optimal experimental parameters for the maximization of the total phenolic compounds extraction were determined. Response values were shown to be mostly suitable to the following regression equation:

PCY = 2.3 + 0.0405T − 0.0006SM + 0.00012T2 + 0.00072S2 + 0.00024TS
(1)

**Figure 1:**
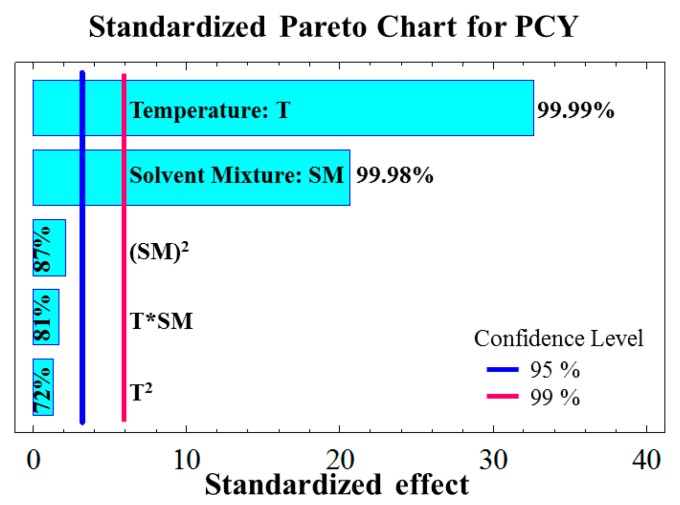
Standardized Pareto charts for phenolic compound yields (PCY); vertical bars blue and red show the confidence level of parameters significance at 95% and 99%, respectively.

The equation expresses the relation between the response variable PCY and the parameters (temperature T and solvent mixture SM) obtained by the application of a multiple regression analysis on the experimental data. Adequacy test showed that a second order regression equation of the model is in complete adequation with the observed results. On another hand, *R*^2^ = 98.91 which means that almost 99% of the experimental results are explained by this model. According to the standardized Pareto charts (Figure 1), PCY is positively affected by the temperature and the solvent mixture (99.99% and 99.98% of confidence level, respectively), which both had a significant linear effect. In consequence the regression equation will be as follow:

PCY = −1.61 + 0.076T + 0.096SM
(2)

**Figure 2:**
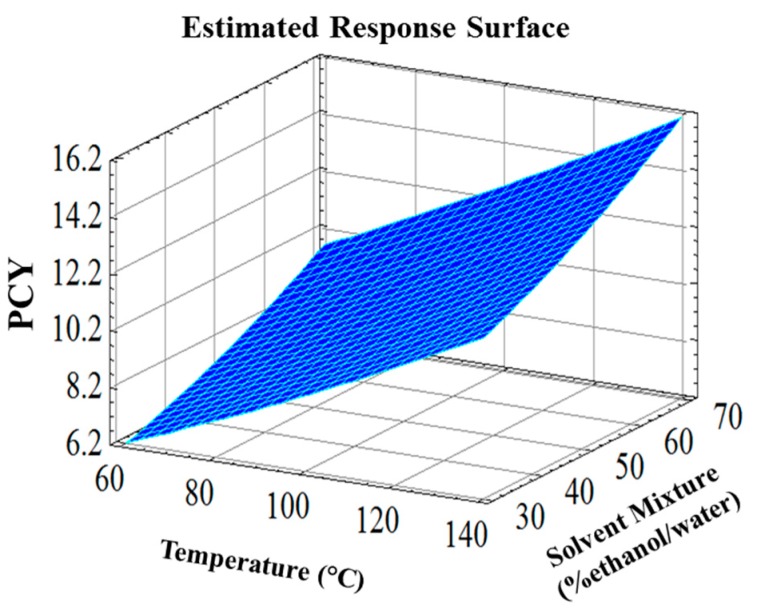
Response surface plot for PCY as function of temperature and solvent mixture, simultaneously.

The response surface plots (Figure 2) showed a three dimensional evolution of PCY as a function of both parameters. Taken together Figure 2 results confirm the linear effect of the parameters with a complete absence of quadratics effects and interaction between parameters.

**Figure 3:**
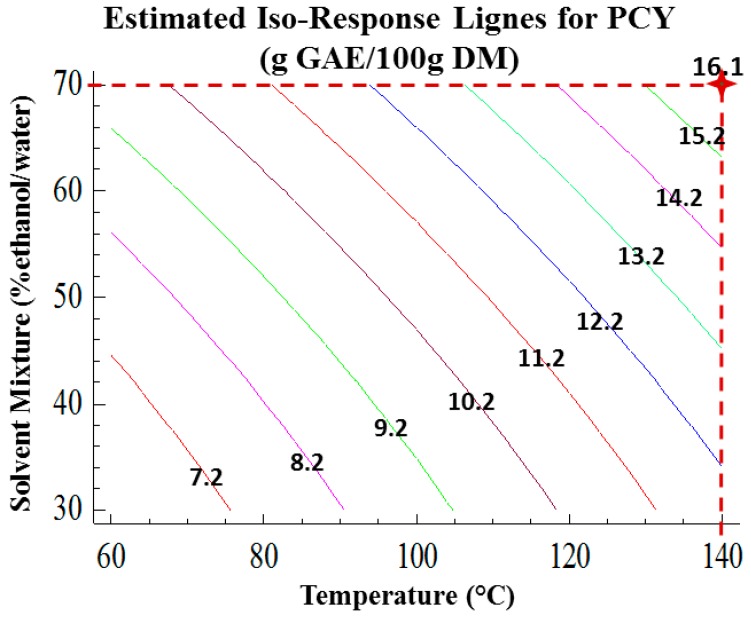
Contours for estimated response surface, showing by the red mark, the optimal extraction parameters for the maximization of the phenolic compounds yield.

The positive influence of the temperature elevation was expected since it increases the mass transfer, ameliorates the solubilization of the solutes in the solvent and diminishes the surface tension and viscosity [8,26]. PCY increased with temperature and reached its peak at 140 °C, which is the highest tested temperature. Many studies showed optimal extraction temperatures of phenolic compounds from different sources varying between 100 and 180 °C [19,27,28,29,30]. In this work, the disadvantages of the increased temperature regarding the quality of the extracted compounds were taken into consideration as well as for energy consumption. Similarly to the temperature parameter, the increase in ethanol content led to a linear increase in the PCY, which attained its maximum with 70% ethanol/water solvent. Figure 3 shows the contours of estimated response surface (Estimated Iso-Response Lignes) of PCY as function of solvent mixture and temperature. It showed that the optimal PCY (16.2 g GAE/100 g DM) is reached with a 70% ethanol/water solvent at 140 °C. These Iso-Response Lignes allow us to choose several combinations between T and SM to reach the same PCY. For instance, extraction at 75 °C in presence of 70% SM or at 120 °C in presence of 30% SM permit to obtain the same PCY of 10.2 g GAE/100 g DM. As compared to Soxhlet extraction, ASE decreases time consumption and solvent use [8]. It has been recognized as a green extraction technique regarding the small amount of organic solvent use [26]. Moreover, at atmospheric pressure conditions, the degradation of anthocyanins occurs at temperatures higher than 50 °C [31], while with ASE it has been elevated to a temperature above 100 °C [27]. A comparative study [29] showed better reproducibility, time efficiency and recovery of Catechins with ASE than with ultrasound-assisted extraction and magnetic stirring. Higher flavonoid recoveries from aspen knot wood were also reported for ASE compared to Soxhlet extraction, sonication and reflux [32]. A linear approach using 30%, 50%, 70% and 90% ethanol/water solvents was conducted on dry grape pomace at 80 °C. Similarly to wet pomace, PCY was the highest for 70% ethanol/water solvent (Data not shown).

### 2.2. Effect of the Raw Material Treatment on the Total Phenolic Content

**Figure 4:**
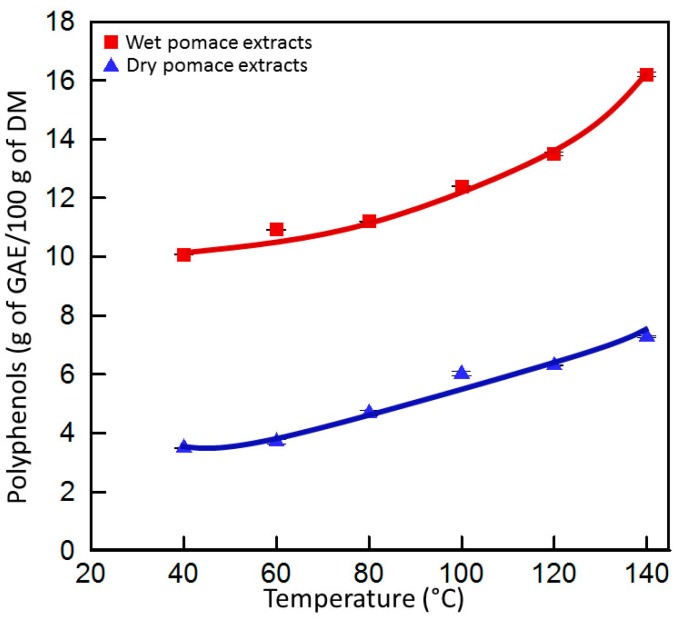
Phenolic compounds content (g of GAE/100 g of DM) of wet and dry pomace extracts at different extraction temperatures in 70% Ethanol/water mixtures.

In this study, the quality of the dehydrated grape pomace was studied. In concordance with our aforementioned results and with the literature review, the temperature elevation enhances the phenolic compounds extraction. This effect is observed for both wet and dry pomace regardless of the raw materials pretreatment. As shown in Figure 4, the phenolic compounds yield extracted with 70% ethanol/water mixture, augments with temperature elevation independently of the raw materials pretreatment and the ascension of both curves reaches maximum values at 140 °C. Our results showed that phenolic compound yields in the wet pomace extracts remain significantly superior to those of dry pomace. This observation seems to be correlated to the drying process. This latter is likely to cause damage to the food altering the physical and biochemical characteristics of the products as well as their color and texture. Degradation of nutritional substances and aroma compounds also occurs [33]. Nevertheless, an augmentation of 1.62 times of phenolic compounds with temperature is observed with wet pomace (from 10 to 16.2 g GAE/100 g DM), and an augmentation of two times is observed for dry pomace extracts going from 40 to 140 °C (Figure 4) (from 3.5 to 7 g GAE/100 g DM) (*p* < 0.05). The extraction process at the lowest studied temperature (40 °C) showed a 2.8 times more PCY for wet (10 g GAE/100 g DM) as compared to dried (3.5 g GAE/100 g DM) pomace extracts. The 24-h drying procedure at 45 °C could have caused the degradation of a non-negligible amount of compounds, thus giving a privileged quantity of phenolic compounds for untreated pomace, which manifests higher yields even at low temperatures. The second reason for this higher phenolic yield observed for wet pomace extracts might be due to their water content. The long period of time in which grape pomace has been subjected to its high water content can be considered as a form of maceration time in which the extraction process has already been enhanced by a pseudo solvent before the actual contact of the pomace with the 70% ethanol/water mixture. The synergistic effect of firstly, the degradation phenomenon of phenolics in dried pomace and secondly, the early enhancement of the extraction process in the wet pomace by its own water content, might lead to 2.9 times more quantity gain in wet compared to dried pomace even at a relatively low extraction temperature. The comparison of the PCY of wet and dry pomace extracts at 40 and 140 °C was conducted. At a low temperature (40 °C), wet pomace have 2.8 times higher PCY than dry pomace, while at a high temperature (140 °C), the amelioration of the PCY diminishes to 2.2 times between dry and wet pomace. Taking into consideration the fact that temperature could affect the hydration of the product; the dry grape pomace phenolic compounds extractability probably increased when temperature increased. Actually, the hydration increases during ASE and in this specific case the product could become less compact and more suitable for solid liquid extraction. In addition to this observation we demonstrated that PCY obtained by ASE from wet pomace was almost 15 times better when compared to the yield we obtained from the same matrices but treated with conventional methods [23].

In conclusion, as expected the optimal parameters for maximizing the accelerated solvent extraction of phenolic compounds are the use of wet grape pomace, in presence of 70% ethanol/water mixture at 140 °C. However, the choice of the optimal extraction conditions should as well take into consideration the biochemical properties of the extracts; therefore, the free radical scavenging properties of the obtained phenolic compounds were subsequently studied. All polyphenol extracts were diluted to 50 micrograms of GAE per milliliter before the radical scavenging activity was studied.

### 2.3. Free Radical Scavenging Properties of the Extracted Phenolic Compounds

The inhibition percentages, representing the free radical scavenging activity of the wet and dry pomace extracts are shown in Table 2.

**Table 2:** 2,2-Diphenyl-picrylhydrazyl (DPPH) free radical inhibition percentage values for the dry and wet pomace extracts (50 µg/mL) compared to Resveratrol and 2,6-di-*tert*-butyl-4-methylphenol (BHT) (50 µg/mL).

	Wet Grape Pomace		Dry Grape Pomace	
Extraction Temperatures	Inhibition Percentage Compared to BHT 50 µg/mL	Inhibition Percentage Compared to Resveratrol 50 µg/mL	Inhibition Percentage Compared to BHT 50 µg/mL	Inhibition Percentage Compared to Resveratrol 50 µg/mL
40 °C	23 ± 1.01	14 ± 0.4	11.4 ± 2.1	11.2 ± 0.2
60 °C	25.9 ± 0.59	16.8 ± 0.8	23.5 ± 0.6	14 ± 0.1
80 °C	30 ± 0.56	21.3 ± 1.4	29.7 ± 0.41	21 ± 0.2
100 °C	35.7 ± 0.78	27.7 ± 0.9	30 ± 0.35	21.8 ± 0.1
120 °C	42 ± 0.89	34.8 ± 1.01	48 ± 2.2	41.5 ± 0.9
140 °C	46.4 ± 1.2	39.8 ± 2.1	54.73 ± 0.44	49.12 ± 1

Both wet and dry pomace extracts showed a concentration dependent free radical scavenging activity. When diluted to 1, 5 and 10 μg/mL, wet and dry pomace extracts had lower inhibition of DPPH radical than that obtained at 50 μg/mL (Data not shown). The inhibition percentage of the wet grape pomace extracts increased with the temperature increase, thus the highest phenolic compounds bioactivity was found for the extracted wet pomace at 140 °C (*p* < 0.05); the inhibition percentage was 46.4 compared to BHT and 39.8 compared to Resveratrol. The same tendency for the free radical scavenging activity increase with temperature elevation was valid as well for the dry grape pomace extracts. The highest inhibition percentage reached at 140 °C was 54.73 compared to BHT and 49.12 to Resveratrol. During the extraction process, the temperature could induce the formation of new components as a result of different occurring reactions. This was considered by some authors as an advantage for the extracts bioactivity [34]. In this study, an average of 2.4 times amelioration of the inhibition percentage was observed for wet grape pomace with the temperature elevation from 40 to 140 °C. Interestingly, a more sharp improvement of the inhibition percentage of about 4.6 times was observed for dry grape pomace within the same temperature elevation interval. When comparing the free radical scavenging activities of wet and dry pomace extracts for all the tested temperatures (Table 2), the inhibition percentages seemed to be divided into two ranges. First, from 40 to 100 °C, the activity was higher for the wet pomace extracts. This could be probably due to an oxidation phenomenon that could likely occur during the drying process, which could probably result in the loss of bioactive molecules and thus leading to an overall lower radical scavenging activity [35]. Second, between 120 and 140 °C where the free radical scavenging effect of the dried pomace extracts became more important when compared to the wet extracts. Following the same aforementioned hypothesis regarding the role of the drying process in the degradation and/or oxidation of the phenolic compounds and the emergence of structurally modified molecules, this treatment could have a positive effect on the overall biochemical properties of the extract, which are only manifested at high temperatures. The main reason for this result could be the selectivity of the drying process, which was carried out at 45 °C overnight. This treatment could eliminate the vulnerable weak bioactive compounds capable of altering the overall free radical scavenging activity and only keeping the highly resistant compounds baring better bioactive properties. These compounds are expected to be less accessible as a part of their resistance to the pretreatment; and it is for this reason that the effect of the dry pomace bioactivity was manifested at 120 and 140 °C. The increase of the antiradical capacity of the grape extracts could be the result of the extraction of high molecular weight polyphenols by a simple thermal treatment above 60 °C. This effect is hidden at low temperatures, during which wet pomace extracts have better scavenging activity, due to the low extractability of those active compounds at low temperatures [36]. Moreover, and during the conventional heat drying process we can talk about a distribution of the product, between the surface and the center. The drying rate is higher when the process starts and falls with the moisture gradient drop, the center of the product do not reach the desired temperature [35]. Therefore, this described process can explain the requirement of high temperatures to extract the remaining highly antioxidant molecules. The potentially vulnerable compounds, oxidized and/or degraded are likely located in the accessible surface of the product. The remaining unaffected bioactive compounds are expected to be located in the center of the dried pomace, protected from air oxidation. Thus, the extraction and accessibility to such compounds would likely require higher temperatures, which can explain further the necessity of high temperatures to show the positive effect of the drying process. On the whole, our results clearly show that ASE extraction with a 70% ethanol/water mixture at 140 °C, provides the highest phenolic compounds yield for wet (16.2 g GAE/100 g DM) and dry (7.28 g GAE/100 g DM) grape pomace extracts. At this same temperature, dry pomace extract demonstrated a better free radical scavenging activity (49.12%) compared to the wet extract (39.79%). Due to the drying process effect on grape pomace, and to the high temperature (>120 °C) extraction process, a specific combination of highly bioactive polyphenols (Gallic and Protocatechuic acids, Gallocatechin, Catechin, Prodelphinidin B3 and Epicatechin) was extracted leading to a positive synergism and a better radical scavenging capacity.

### 2.4. Characterization, Identification and Quantification of Phenolic Compounds by HPLC-DAD

**Figure 5:**
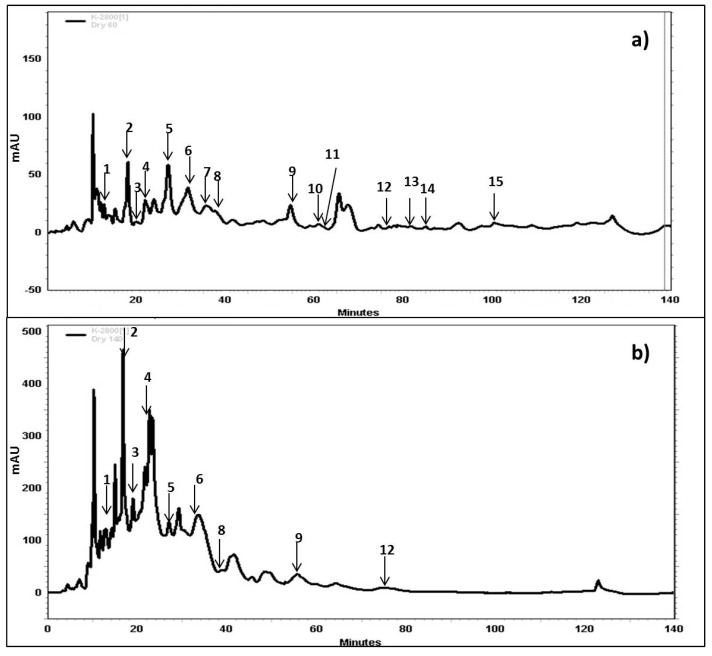
Representative HPLC chromatograms of phenolic compounds extracts of dry grape pomace obtained at 60 °C (3.9 g GAE/100 g DM) (**a**) and 140 °C (7.3 g GAE/100 g DM) (**b**) detected at 280 nm UV wavelength. Peaks: 1, Gallic acid; 2, Protocatechuic acid; 3, Prodelphinidin B3; 4, Gallocatechin; 5, 4-Hydroxybenzoic acid; 6, Catechin; 7, Caffeic acid; 8, Epigallocatechin; 9, Epicatechin; 10, Ferulic acid; 11, Gallocatechin gallate; 12, Resveratrol; 13, Rutin; 14, Cinnamic acid; 15, Kaempferol.

In the literature, HPLC-DAD detection is a common tool used to the determination of phenolic compounds in food matrices. In our study, and in order to characterize the phenolic extracts obtained from either wet or dry grape pomace, an identification followed by a quantification of several phenolic compounds were conducted using HPLC-DAD. The chromatograms showed different profiles for the analyzed samples when considering the extraction temperatures and the raw material water content. The phenolic compounds content of the extract (dry pomace, 140 °C) with the highest free radical scavenging activity contained the highest Gallic acid quantity (0.026 g/100 g DM) amongst the 12 extracts. It also contained important yields of Gallocatechin (0.45 g/100 g DM), Prodelphinidin B3 (0.055 g/100 g DM), Catechin (0.09 g/100 g DM) Protocatechuic acid (0.5 g/100 g DM) and Epicatechin (0.024 g/100 g DM) thus demonstrating the important yields of flavonoids molecules found in the dry extracts at 140 °C compared to non-flavonoids especially Resveratrol which was barely detected in all extracts (Figure 5b). Based on these results and in agreement with previous literature findings; we can associate this high bioactivity to the Gallic acid and all flavonoids molecules found in the 140 °C extract [37,38,39]. Compared to dry pomace extract at 140 °C, wet pomace extract at 140 °C contained less Gallic acid (0.011 g/100 g DM) and Prodelphinidin B3 (0.055 g/100 g DM), no Catechin, Epicatechin or Protocatechuic acid. Moreover, wet pomace extract at 140 °C contained Ferulic (0.013 g/100 g DM) and Chlorogenic (0.004 g/100 g DM) acids.

**Figure 6:**
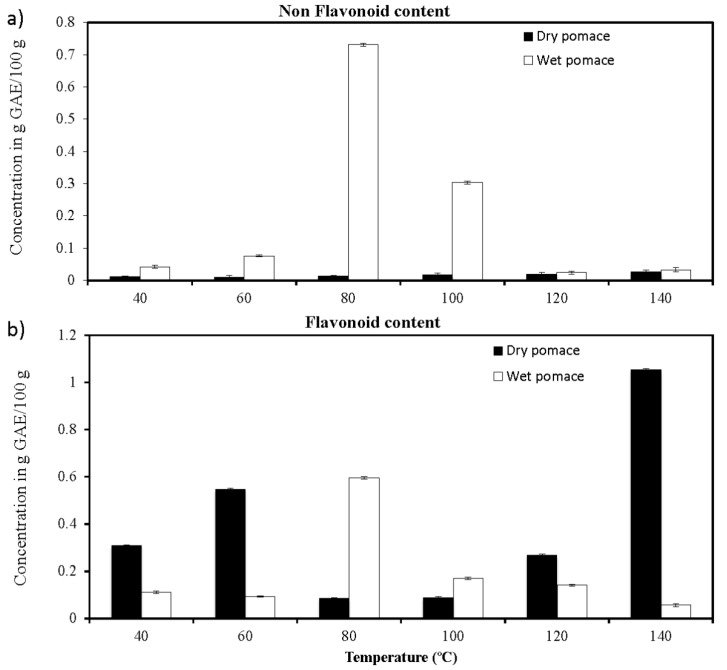
Histograms representing the quantification of non-flavonoids (**a**) and flavonoids (**b**) molecules extracted from dry and wet grape pomace at 40, 60, 80, 100, 120 and 140 °C as obtained by HPLC-UV analysis.

The presence of high Prodelphinidin B3 content in this extract highlights the heat-induced extraction of bioactive high molecular weight polyphenols. The remarkable quantities of Prodelphinidin B3, which are polymeric tannins, could also be in direct relation with the high radical scavenging activity observed [40]. Moreover, low molecular weight compounds, such as Gallic acid (Figure 6b, peak 1), Protocatechuic acid (Figure 6b, peak 2) and 4-Hydroxybenzoic acid (Figure 6b, peak 5) were also found in the extracts probably contributing to the high radical scavenging activity. In comparison to the other temperature conditions used in the extraction process, we show as an example the chromatogram obtained for the dry byproduct sample extracted at 60 °C (Figure 5a). These chromatograms clearly show the enhancement of the phenolic compounds yields as a function of temperature. This tendency was as well observed in all other chromatograms obtained at 40, 60, 80, 100, 120 and 140 °C either for the wet samples or the dry ones (Data not shown). However, the identified phenolic compounds peaks represented in Figure 6 do not follow the same tendency of the total phenolic compounds, because a limited number of polyphenol standards were identified.

The overall interpretation of the HPLC results and the quantification of the phenolic standards yields found in all extracts were realized by comparing the two major classes (non-flavonoids and flavonoids) of PC (Figure 6a,b). In general, non-flavonoids molecules were practically absent from all extracts except the ones obtained at 80 and 100 °C from wet pomace. As to flavonoids molecules, these were by far more present in the extracts especially those obtained at 80 °C from the wet pomace and at 60 and 140 °C from the dry pomace. No direct correlation between the flavonoid content and the free radical scavenging activity was clearly found (Table 2). Some studies associate non-flavonoid content such as Resveratrol and Gallic acid in the enhancement of scavenging activity, while others have demonstrated the implication of flavonoid compounds in the biochemical properties exhibited by phenolic compounds [38,41]. Indeed this is what we can conclude from our results where the best inhibition percentages of DDPH were observed when using extracts obtained from dry pomace at relatively high temperatures, namely 120 and 140 °C (Table 2). These extracts are relatively rich in flavonoids as shown in Figure 6b. The importance of our results is that the best inhibitions were obtained when flavonoids molecules such as Gallocatechin, Prodelphinidin B3 and Catechin were accompanied in the extracts by non-flavonoids molecules such as Gallic acid, Protocatechuic acid and 4-Hydroxybenzoic acid. We can suggest that the combination of these molecules induces a synergistic effect, which is the basis of the free radical scavenging activity highlighted in our work. This synergistic effect, between different kinds of flavonoids and non-flavonoids molecules, has been previously shown to be implicated in the antimicrobial properties exhibited by phenolic extracts obtained from different *Vitis vinifera* cultivars [42].

## 3. Experimental Section

### 3.1. Reagents

All reagents were of analytical grade. Folin-Ciocalteu reagent; Sodium Carbonate; 2,6-di-*tert*-butyl-4-methylphenol (BHT); 2,2-diphenyl-picrylhydrazyl (DPPH) radical and Tris-HCl buffer were obtained from Sigma Chemical Co. (St. Louis, MO, USA). Solvents used for high-performance liquid chromatographic analysis were Methanol (Merck, Darmstadt, Germany), Formic acid (Scharlau, Barcelona, Spain) and water (Scharlau, Barcelona, Spain) of HPLC ultra gradient grade. Phenolic compounds standards of Gallic acid, Protocatechuic acid, *p*-Coumaric acid, Caffeic acid, Ferulic acid, Cinnamic acid, Chlorogenic acid, 4-Hydroxybenzoic acid, Catechin, Epicatechin, Epigallocatechin, Gallocatechin, Catechin gallate, Gallocatechin gallate, Prodelphinidin B3, Resveratrol, Kaempferol, Quercetol, Myricetol and Rutin were from Sigma Chemical Co. (St. Louis, MO, USA).

### 3.2. Sample Preparation

The Cabernet Sauvignon grape pomace was provided by Château KSARA (Beqaa Valley, Lebanon), after the harvesting season of September 2011. Grape pomace containing seeds and skins, was packed and stored at −20 °C until analysis. The drying process was performed at 45 °C overnight with a simple layer and under a laminar airflow drying oven. Wet and dry grape byproducts were milled to a 2 mm particle size.

### 3.3. Dry Matter Content

The dry matter content for the raw material was carried out by weighing an appropriate amount of sample and dried during 24 h in a ventilated oven at 105 °C. The dry matter content in the wet grape pomace was 49.1% ± 0.9% and in the dried pomace 94.3% ± 0.2%.

### 3.4. ASE Extraction

Accelerated Solvent Extraction of phenolic compounds was performed using a Dionex ASE 100 extraction system (Dionex, Sunnyvale, CA, USA) at 100 bars pressure. 38 g of wet or dry milled pomace were placed into a 100 mL extraction cell. The ASE variables, static time (incubation time between the solvent and the grape pomace inside the extraction cell) (0 min), flush volume (the volume of solvent to flush through the extraction cell after heating step to the required temperature) (100%), purge time (the time in which the cell is purged with nitrogen) (120 s) and static cycles (number of times in which the heating and flushing processes occurred) (five) were used as basic conditions. The choice of the ASE variables was done based on preliminary studies (Data not shown). Grape pomace is therefore rinsed by the passage of the heated solvent through the extraction cell. Thus, the extraction process takes place during the passage of the extraction solvent through the cell with a 0 min static time. Under these conditions the total duration of the accelerated solvent extraction process is about 15 min. With 100% flush volume and 100 mL extraction cell, the volume of the collected extract was equal to 100 mL. Several runs of dry and wet milled pomace were performed, testing several ethanol/water ratios (30%, 50%, and 70% ethanol/water) and their capacity to extract phenolic compounds from grape pomace. The effect of several extraction temperatures (40, 60, 80, 100, 120, and 140 °C) on the recovery of the total phenolic compounds was also studied. The ethanol/water ratio and ASE temperatures were chosen based on preliminary studies. After each run, the extract was mounted on a rotary evaporator (Laborota 4000-efficient, Heidolph, Schwabach, Germany) to diminish its final volume and concentrate polyphenols. Water and the organic solvent (ethanol) were evaporated at 40 °C in a heated water bath. After the evaporation process, extracts volumes were adjusted to 50 mL (for dry pomace) and 26 mL (for wet pomace) with distilled water. Since the dry matter content of dry pomace (94.3%) is 1.92 times higher than that of wet pomace (49.1%), the final volume of dry pomace extracts (50 mL) was adjusted to be 1.92 times higher than that of wet pomace extracts (26 mL) and that for subsequent simplification of the calculation process. The extracts were then transferred to plastic vials and stored at −20 °C until analyzed.

### 3.5. Total Phenolic Compounds Determination

According to the Folin-Ciocalteu method previously described by Slinkard and Singleton (1977) [43], an aliquot of 10 μL of the sample solution was mixed with 100 μL of commercial Folin-Ciocalteu reagent and 1580 μL of water. After a brief incubation at room temperature (5 min), 300 μL of saturated sodium carbonate was added. The color generated was read after 2 h at room temperature at 760 nm using a UV-Vis spectrophotometer (UV-9200, BioTECH Engineering Management, UK). The Phenolic Compounds Concentration (PCC) of the samples was quantified and expressed as milligrams of Gallic Acid Equivalent (GAE) per liter (mg GAE/L). Phenolic Compounds Yield (PCY) derived from these values was given by transforming mg GAE/L into grams of GAE per 100 g of grape pomace dry matter (g GAE/100 g DM).

### 3.6. Free Radical Scavenging Activity

According to the modified method of Gyamfi *et al.* (1999) [44], the free radical scavenging activity was measured by the capacity of the phenolic compounds contained in the samples to reduce DPPH (2,2-diphenyl-picrylhydrazyl), a stable free radical. The total phenolic compound concentration of the wet and dry extracts, found by Folin-Ciocalteu in mg GAE/L was diluted to 50 μg GAE/mL before measuring the radical scavenging capacity of the extracts. 50 µL of various sample extracts concentrations (50 μg/mL) or positive control (BHT and resveratrol) diluted in pure ethanol were added to 450 µL of Tris-HCl buffer solution (50 mM, pH 7.4). 1.5 mL of DPPH Solution (0.1 mM) were added to the mixture. Absorbance at 517 nm was measured after 30 min of incubation at room temperature using pure Methanol as a blank. The inhibition percentage of the DPPH free radical is calculated as follows: Inhibition Percentage = [(absorbance of standard − absorbance of sample)/absorbance of standard] × 100.The free radical scavenging activity of extracts was examined by comparison with that of known antioxidants such as Butylated Hydroxytoluene (BHT) (a synthetic antioxidant) and Resveratrol (a natural antioxidant).

### 3.7. HPLC Analysis:

Phenolic compound analyses of the extracts prepared from grape pomace were performed by high-performance liquid chromatography (HPLC). Prior to analytical chromatography, samples and standards were purified by filtration through 0.2 μm syringe filters (Polyethersulfone membrane). Equipment consisting of a liquid chromatography apparatus (KNAUER, Berlin, Germany) coupled to a diode array detector was employed. Analyses were performed on a Spherisorb ODS-2 (5 μm, 250 × 4.6 mm) column, at a flow rate of 1 mL·min^−1^, using a 20 μL injection volume and a detection at 280 and 320 nm. Eluent A was 2% aqueous Formic acid and eluent B: 69% Methanol (MeOH), 29% HPLC grade water and 2% Formic acid. The elution program was: 100% solvent A for the first 3 min, 90% A from 10 to 60 min, 60% A from 60 to 80 min, 40% A from 80 to 105 min, 20% A from 105 to 120 min, 0% A from 120 to 140 min to reach 100% A at 140 min. Identification was based on comparing retention times of the peaks detected with those of original compounds, and on UV-Vis online spectral data. Quantification was accomplished using the phenolic standards solutions. Results were expressed as mg/mL of grape pomace extract volume [45]. All chromatograms were treated using ChromGate (version 2.8.) software. All peaks were identified and quantified using the corresponding phenolic compounds as internal standards. Peaks overlapping was taken into consideration by the software during the calculation.

### 3.8. Experimental Design

A three-level central face composite design was developed to assess the main impact of two factors: solvent mixture and extraction temperature as well as their interaction on total phenolic compounds yield. The extracts were obtained from Cabernet Sauvignon grape byproducts by Accelerated Solvent Extraction. Temperature values varied between 60 and 140 °C and solvent mixture between 30% and 70% ethanol/water. Both temperature and solvent mixture intervals used in the study were deduced from preliminary trials realized previously. The two independent variables were coded at three levels (−1, 0, 1) resulting in an experimental design of nine experimental points. Considering two parameters and one response, experimental data were fitted to obtain a second order regression equation of the form:

Y = β0 + β1T + β2SM + β12T.SM + β11T2 + β22S2
(1)

where Y is the predicted response parameter, T is the temperature, SM is the solvent mixture, β0 is the mean value of responses at the central point of the experiment; β1 and β2 are the linear coefficients, β11 and β22 the quadratic coefficients and β12 the interaction coefficient. Experimental design and statistical treatment of the results were performed using STATGRAPHICS Plus 4.0 for Windows. The software was used to generate response surfaces and contour plots.

### 3.9. Statistical Analysis

Analyses were repeated at least three times. Means and error bars are represented on the figures. Analyses of variance (ANOVA) were conducted to estimate the significant parameters (linear, quadratic and interaction between parameters).

## 4. Conclusions

The objective of this study was to design an experimental procedure for the extraction of phenolic compounds from grape byproducts through accelerated solvent process taking into consideration the effect of the drying pretreatment on the overall yield of phenolic molecules and their antiradical properties. The optimal extraction parameters were found to be 70% ethanol/water mixture at 140 °C. The drying pretreatment of the raw material affected significantly the yields and the bioactive properties of the resulting compounds. It was clearly shown that the diversity and the content of the extracted phenolics were directly related to the pretreatment of the grape pomace, either wet or dry, and to the extraction temperature. The drying process seemed to have a negative effect on the accelerated solvent extraction of polyphenol from grape pomace at temperatures lower than 100 °C. Beyond this temperature, and up to 140 °C, the polyphenols extracts from dry pomace exhibited higher radical scavenging activity due to a particular polyphenol mixture and thus to a clear synergistic effect between different phenolic compounds. Accelerated solvent extraction seems to be a promising method for the preparation of highly concentrated and bioactive phenolic extracts, especially that medium pressure solvent extraction technology is available on an industrial scale. This valuable technology could be applied in different food industries to valorize byproducts through the extraction of several phenolic compounds using a cost-effective process and an environmentally friendly solvent.
